# Training Program to Modify Manual Wheelchairs to Simplified Power Wheelchairs for Community Dwelling Elderly People and Caregivers

**DOI:** 10.1155/2022/5594598

**Published:** 2022-03-10

**Authors:** Suchitporn Lersilp, Supawadee Putthinoi, Theeratorn Lersilp, Kewalin Panyo, Autchariya Punyakaew

**Affiliations:** ^1^Department of Occupational Therapy, Faculty of Associated Medical Sciences, Chiang Mai University, Thailand; ^2^Department of Special Education, Faculty of Education, Chiang Mai Rajabhat University, Thailand

## Abstract

Mobility aids, particularly power wheelchairs, are necessary for elderly individuals who have health problems and disabilities. However, there is a limitation in providing power wheelchairs for such people in the community. The objectives of this study were to develop a prototype for a simplified power wheelchair and develop and evaluate a training program that has the potential to encourage evaluation and modification of the wheelchair for the elderly and their caregivers in the community. Twenty-four participants consist of elderly people and caregivers who were interested in the training program that comprised two sessions: theory and fieldwork experience. Results showed that the elderly people and caregivers, who had no knowledge or experience of wheelchair modification, were able to learn and provide suggestions for wheelchair users in their community. Two themes emerged from evaluating the training program, which included “benefits from the training program” and “improvement of the training program in the future.” Key concepts were elicited and considered in six categories: sufficient knowledge and practical learning to build confidence, values of fieldwork experiences, team support, organizational support, expansion of various contexts, and system of continued connection and services after training.

## 1. Introduction

National statistics from 2012 have indicated that 1.5 million people have disabilities in Thailand [1]. From this population, the elderly held a higher percentage of disabilities (9.8%) than other age groups. Moreover, a higher percentage of elderly people with disabilities lived in suburban and rural communities rather than in urban areas. Furthermore, the ratio between childhood age, working age, and the elderly population is expected to change from 50 : 100 : 20 in 2010 to 23 : 100 : 47, respectively, in 2040 [2, 3]. This reflects an increase in the number of elderly people in Thailand, which is moving toward an aging society. This situation is being caused by a rapid acceleration of low birth rate and the tendency of longer life in elderly people. Whenever elderly people live longer without healthcare or social support, they face a high risk of health problems and disabilities [4]. They also experience many age-related changes that have impact on their functions and performance, especially musculoskeletal functions. These changes have greater impact on elderly individuals with disabilities [5–7]. This situation does have impact not only on elderly individuals but also on their families. Some family members give up their jobs to become caregivers for disabled elderly relatives. Leaving their jobs creates loss of income, abandoned social participation, stress, and low self-esteem [4, 8]. Supportive services for the elderly with disabilities should be aware of how their families are also affected. In other words, elderly individuals have physical problems that limit mobility, and their caregivers have cultural behaviors and expectations, and so both factors have impact on occupational engagement.

Assistive technology, particularly a mobility aid, is an optional support service for elderly individuals with disabilities because the main problem is musculoskeletal function. This problem creates the limitation of mobility, which reduces the opportunity for participating in social and community activities [9–11]. However, these individuals can be encouraged to perform their daily life activities independently, which decreases fatigue, stress, and anxiety among the caregivers. Nevertheless, the national survey from 2002 to 2007 indicated that only 28.6% of the people with disabilities use assistive technology [3]. The reasons for this were that many individuals with disabilities in communities had a long wait for provision of assistive devices, and some of them felt uncomfortable in using unsuitable equipment [8, 12, 13]. In other words, the assistive devices sometimes did not fit the users' needs or they had limitations. This might be due to the Rehabilitation Act, in which individuals with disabilities were provided with only basic devices, such as a crutch, cane, walker, or manual wheelchair, while some individuals had severe problems that required more advanced technologies.

The wheelchair is assistive technology that is provided usually for people with disabilities, especially elderly individuals. Although it is an aid in mobility that supports the independent movement of elderly individuals and conserves energy for caregivers, it must be custom fit for the users' varied needs and abilities [14]. Previous studies indicated whether the wheelchair was fit for reducing injury, increasing its effectiveness, and promoting quality of life [15–18]. Elderly individuals with disabilities, in particular, not only were disabled but also lost physical or cognitive functioning for which basic devices did not help to meet their needs. Besides, physical needs related to differences in wheelchair use and the environment were important keys that had impact on the user [19]. In Thailand, the provision of wheelchairs for the elderly in the community still has limitations, as its management system is administered generally by the subdistrict municipality and a local community hospital. The typical process of provision would start with the community development officers of the subdistrict municipality by proposing lists to the provincial social development and human security office of registered individuals with disabilities, who meet the government criteria. However, this procedure takes a long time. Some individuals actually died before being given a wheelchair [13], while others with medium to high socioeconomic status used personal funding to buy their own wheelchairs.

At times, manual wheelchairs might not be enough to enhance performance or help in accessing daily life activities. The power wheelchair is a high-tech assistive device that would be a choice for elderly individuals with disabilities to consider. Power wheelchairs have been continuously developed to improve their effectiveness for users to perform their daily activities easily [20]. Although the power wheelchair tends to satisfy its users, it is used rarely in hospitals and not provided for low-income users [17]. Most elderly people, particularly in communities, have low to medium income, and local government has not been able to support them. Because the manual wheelchairs are inexpensive, they are generally provided by the government. Attempts have been made to relate service providers to adaptation and modification of the manual wheelchair for the purpose of suitability for users and their increased quality of life [21, 22]. In Thailand, many elderly individuals in communities were provided with wheelchairs through the lending system of the local community hospital or personal donation [13]. This was due to the Thai belief that it would be bad luck if mobility aids were left unused in the home. Thus, when users died or recovered from temporary dysfunctions, their mobility aids would be donated to the local community hospital or other people who needed them. Therefore, many manual wheelchairs have been donated to the local hospital in the community and centers for village community health. However, there were two main problems in this provision. First, wheelchairs in the lending system of the local community hospital were neither evaluated for their suitability for each user nor checked for their condition after being returned. Second, most of the people who borrowed the wheelchairs were caregivers or relatives, who had never been trained to use and maintain them. Many of the wheelchairs returned to the hospitals were broken and unfit to pass on to other users [8, 13].

This research study focused on developing a training program that covered the development of a prototype for a simplified power wheelchair set, with a low-cost option for elderly individuals, when a standard manual wheelchair did not meet their needs. In addition, the program considered the characteristics of elderly learners and their caregivers who had no previous knowledge or experience of wheelchairs. This would maintain the potential to encourage the elderly and their caregivers to participate in community activities, as worthy community members. Finally, wheelchair evaluation and modification in the training program are still a novelty for elderly people and their caregivers in the community. Perspectives from evaluating all aspects of the training program were useful for local policymakers and health service providers at the primary care level. Therefore, the objectives of this study were to develop a prototype for a simplified power wheelchair and develop and evaluate a training program that has the potential to encourage the elderly and their caregivers in the community to evaluate and modify the wheelchair.

## 2. Material and Methods

This study was a mixed-methods designed project approved by the Research Ethics Committee of the Faculty of Associated Medical Sciences, Chiang Mai University, Thailand (AMSEC-61EX-019). After announcing the research project to community villages, there were 24 participants who were elderly people and caregivers interested in the study and who signed research consent forms. They were selected from a suburban community of 12 villages by using a purposive sampling method. The inclusion criteria were as follows: elderly people or caregivers who were interested in evaluating and modifying the wheelchair, were able to read and write in the Thai language, and had no severe visual, hearing, or hand problems. The exclusion criteria comprised severe disabilities in learning and inability to participate in any of the sessions of this study.

The method of this study comprised two phases as follows:

Phase 1: Development of a Prototype for a Simplified Power Wheelchair Set

This phase involved working with medical engineers in order to design and develop a prototype that was simple for the participants to study and handle.

Phase 2: Development and Evaluation of a Training Program to Encourage Potential Evaluation and Modification of a Manual Wheelchair to a Simplified Power One for Elderly People and Caregivers in the Community

### 2.1. Development of the Training Program

**Figure 1:**
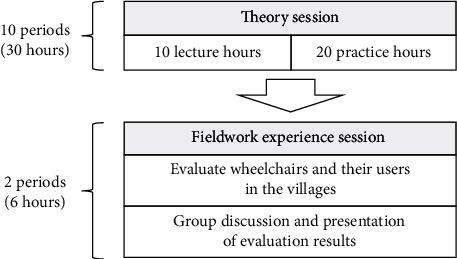
Process of the training program.

This phase involved designing, processing, and examining the training program, which was suitable also for the background and characteristics of the participants. This training program was developed and adjusted from the World Health Organization (WHO) Wheelchair Education Training Package-Thai version [23] and basic knowledge of the wheelchair, which related to simplifying the power wheelchair and analyzing the limits of understanding, experience, and learning strategies of the elderly people. Instruments for this phase comprised a manual on the training program, manual wheelchairs, a general mechanical repair tool set, tape measures, a fieldwork worksheet, and the prototype for the simplified power wheelchair, which was the output from the first phase. There were two sessions of theory and fieldwork experience, as shown in Figure 1.

The session on learning theory consisted of 10 periods totaling 30 hours, including 10 hours of lecture and 20 of practice. Six topics were reviewed in this session and included basic knowledge of assistive devices; types and functions of the wheelchair; parts of the wheelchair; consideration of the wheelchair for elderly individuals with disabilities; adjusting and repairing uncomplicated problems with the wheelchair; and modifying the manual wheelchair to a simplified power one and maintaining it. Each period contained a 1-hour lecture and 2 hours of practice. In the 1-hour lecture, the participants were taught in a relaxing environment with explanations using informal language, demonstration, examples of case studies, role play, and questions and answers while learning. In the hours of practice, the participants learned in a practical work group, with varied learning activities such as following steps in practice, taking part in collaborative games, solving challenging problems, discussing, and presenting.

**Figure 2:**
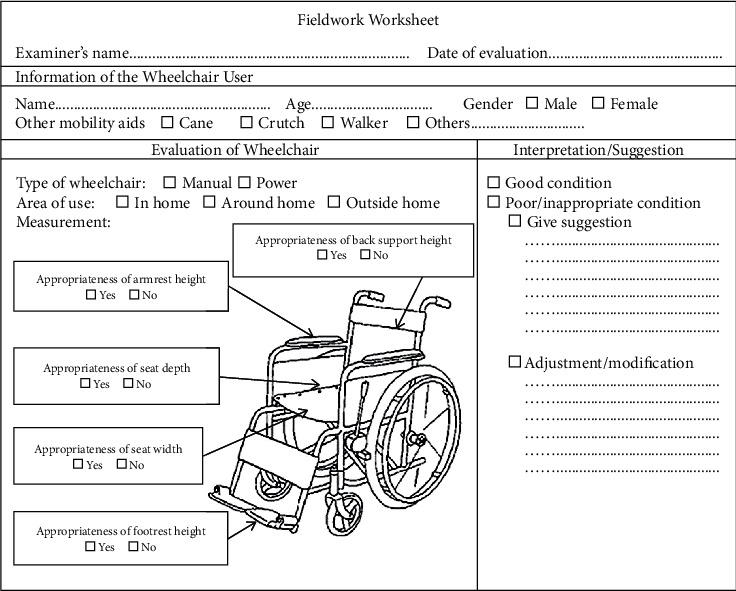
Fieldwork worksheet.

The theory session took place before the fieldwork experience one. In the theory session, the participants were trained to use the fieldwork worksheet as a tool in this step, which was developed by researchers for helping the participants to use the wheelchair easily, as shown in Figure 2. In the fieldwork experience session, the participants received a group work assignment, with each group consisting of 3 participants, who evaluated the wheelchairs and made suggestions, followed by dealing with user problems with the wheelchair. Each group would keep in contact, give information on the training program, and make appointments with wheelchair users in their village.

### 2.2. Evaluation of the Training Program

The evaluation was divided into two periods. The first one was carried out in the form of individual oral and practical examination after finishing the theory session. Each participant had to explain parts of the wheelchair and their functions, analyze uncomplicated problems of the wheelchair, and demonstrate modifying the manual for a simplified power wheelchair. After finishing the fieldwork experience session, the second period was performed by using focus group discussion conducted by the researchers, with the purpose of exploring perspectives of the participants regarding the efficiency of the training program. The participants were separated into two groups: Focus Group A (FG-A-1 to FG-A-12) and Focus Group B (FG-B-1 to FG-B-12). Each group consisted of 12 participants. The representative status of Focus Group A was elderly people while Focus Group B was for the caregivers, which comprised family members and community volunteers.

Each focus group discussion had two researchers, who took on the roles of group facilitator and moderator. Due to the different backgrounds of the participants, the facilitator worked with the moderator in order to encourage the participants to share their perspectives in a relaxed environment. Two research assistants were assigned to record data from the discussion by audio-recorder and notetaking. Duration of the focus group discussion was around 60 minutes, which included 3 steps as follows.

- Presession

The researchers explained the objectives of the discussion and built a comfortable and relaxed atmosphere for sharing opinions and feelings.

(ii) Discussion session

The discussion was derived from three probe questions.

- Is the training program useful for you as an elderly person or a caregiver? If yes, please give your opinion regarding how useful the training program was for you. If no, please give your opinion on what problems prevented the training program from being useful to you
- Is the training program suitable for elderly people and caregivers in communities? If yes, please give reasons. If no, please suggest how to improve the training program for the future
- Overall, are you satisfied with this training program? If yes, please give your opinions regarding its strengths. If no, please indicate the points that you are dissatisfied with

(iii) Conclusion session

The researchers summarized the opinions and perspectives gathered, and the participants clarified, confirmed, or elaborated on the summarized information.

Thematic analysis was carried out in order to analyze the qualitative data that were gathered from the focus group discussion.

## 3. Results

Most of the participants were male (75%). The ages of the study participants ranged from 39 to 77 years, a range of 38 years. The average age was 60.80 ± 9.79 years. In addition, a majority of them graduated at secondary education level (33.33%), followed by primary education level (29.17%), and high school level (25.00%). Only one from the 15 elderly people was a government retiree, who still had income from the government, while the remainder had stopped their employment and had unpredictable incomes. The caregivers had temporary jobs and were hired with unpredictable incomes. Half of the participants (50.00%) had incomes below 3,000 baht per month. Study participants consisted of elderly people and caregivers. However, 3 participants were elderly people who played the role as family caregivers. Moreover, all of the participants had no experience in evaluating, using, training for, fixing, or maintaining a wheelchair or any other mobility aids. The characteristics of the participants are shown in Table 1.

### 3.1. A Prototype for a Simplified Power Wheelchair Set for Elderly People and Caregivers in the Community

The researchers worked with a medical engineer to design the prototype with specific functions. The design was based on five considerations as follows: First, the prototype should be constructed with few main parts in order to prevent confusion among the learners. Second, the power should be enough to drive a regular manual wheelchair on a flat surface, a slope, and a rough road or walkway. This was because most of the communities were located in suburban areas, where thoroughfares were not always smooth and sometimes a dirt road. Third, the prototype should be assembled with general mechanical tools that can be found readily in the community. Fourth, all parts had to be removable and uncomplicated to set up and remove. Fifth, the cost of modification should be lower than the general market price for power wheelchairs, with the possibility of personal payment or funding from community support.

**Figure 3:**
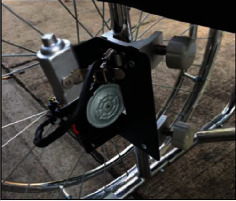
The electric motor of the prototype for the simplified power wheelchair.

**Figure 4:**
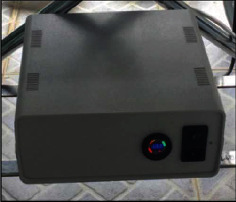
The control box of the prototype for the simplified power wheelchair.

**Figure 5:**
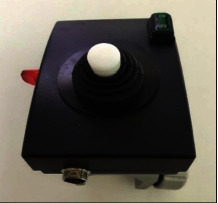
The direction controller of the prototype for the simplified power wheelchair.

After considering the scope of design, the prototype was comprised of three parts including an electric motor, a control box, and direction controller; mechanical assembly required only four steps. In terms of the prototype parts, the electric motor helped the manual wheelchair to move while carrying the user. Although a high-powered electric motor could help to move a wheelchair faster, the elderly might find it difficult to control, particularly those who have problems with controlling a motor or with delayed reactions. As a result, the lowest electrical power sufficient for driving on a flat surface, a slope, and rough road or walkway was the Motor Gear 12VDC 100 W 6 Ah (Ampere-Hour), with two reduction gears, as shown in Figure 3. The control box, as shown in Figure 4, was the electric power sending part connected between the electric motor on the left and right wheels and direction control. In this study, the DC Motor Drive 12 V 40A with 1 Ah electric power was chosen and set up in the control box. A 12 V 18 Ah battery, which had a full load for 2 hours continual driving, was contained in the control box. In fact, the users hardly ever used the wheelchair continuously for long periods of time; therefore, the battery could last long enough for completing general daily activities, with recharging once a day. The direction controller, as shown in Figure 5, was handled by the joystick (Axis Analog Output) (100 K) and could move in four directions; forward, backward, left, and right. The function of breaks was included in the direction controller. The user was able to release on the controller to stop the manual wheelchair with the prototype assembled.

**Figure 6:**
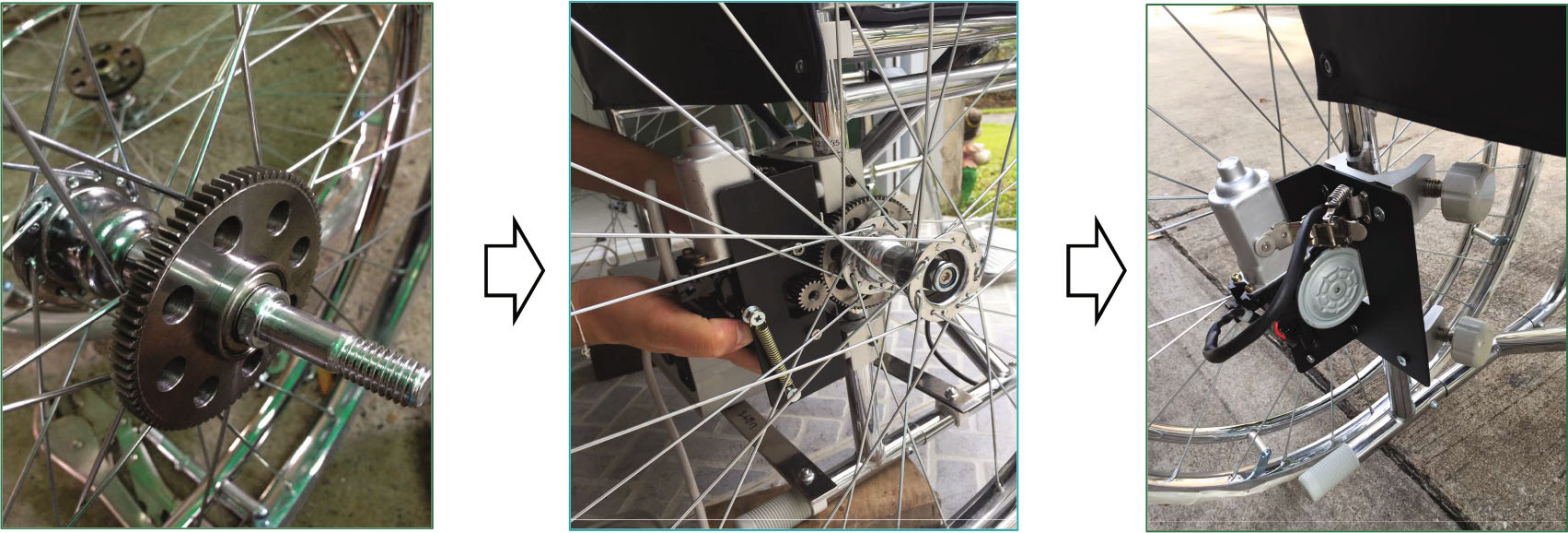
Setting up the electric motor.

**Figure 7:**
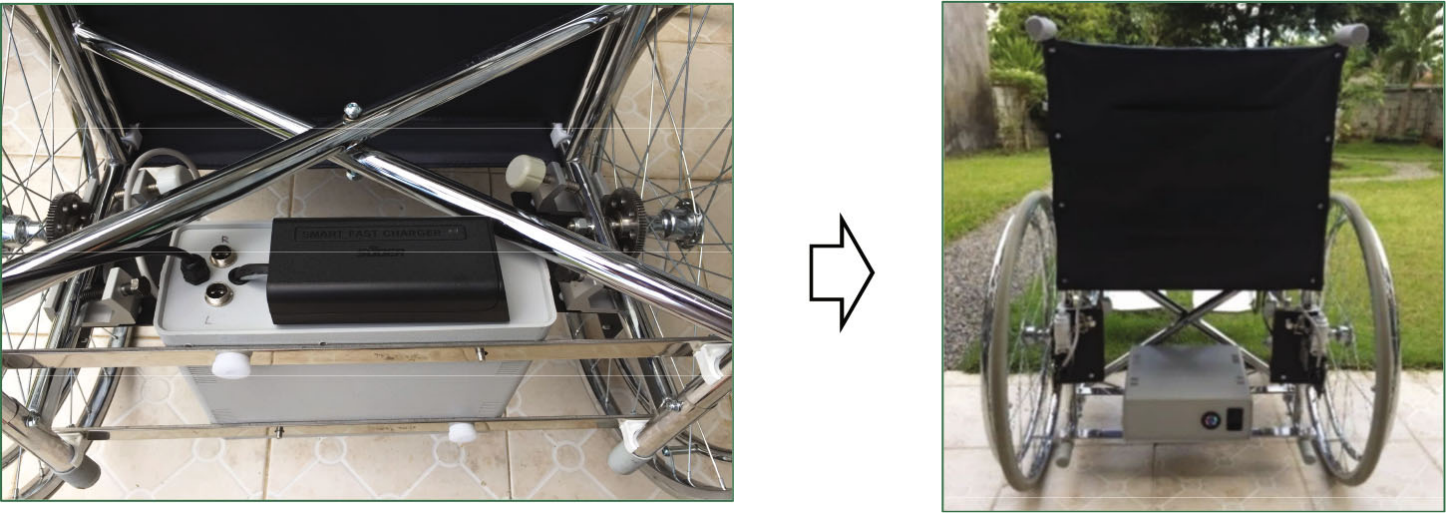
Setting up the control box.

**Figure 8:**
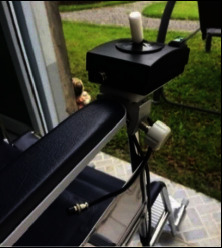
Setting up the direction controller.

**Figure 9:**
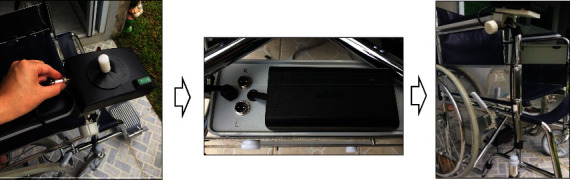
Connecting all of the main parts with a set of electric wire.

There were four steps of mechanical assembly: setting up the electric motor, control box, and direction controller and connecting all main parts by electric wire as follows:

- Setting up the electric motor started by removing the wheels and wheel bearings. Next, the main gear was put on the wheel axle, and the wheels were returned to their normal position. Then, the electric motors were put on the inner sides of both wheels and held by two locked gears, as shown in Figure 6
- Setting up the control box started by installing the stainless-steel bars used to mount the control box to the wheelchair frame. Then, the control box and bars were held by screw nuts, as shown in Figure 7
- In setting up the direction controller, screw nuts held it on an armrest of the wheelchair, as shown in Figure 8, in which the armrest was used, left or right, depended on the side of the dominant, or unaffected hand of the users
- All of the main parts were connected by a set of electric wire. After connecting the jacks to the connectors, the electric wire should attach them together with the frame of the wheelchair, as shown in Figure 9

### 3.2. The Training Program to Modify the Manual Wheelchair to a Simplified Power One for Elderly People and Caregivers in the Community

After finishing the theory session, most of the participants (91.67%) were able to explain and demonstrate modification of the manual wheelchair to a simplified powered wheelchair without receiving any verbal cues. The remaining participants (8.33%) needed general verbal cues to analyze uncomplicated wheelchair problems and also specific feedback on modifying the manual wheelchair to a simplified power one.

Next, the focus group discussion was performed to evaluate the training program, of which its main results revealed two themes: “benefits from the training program” and “improvement of the training program in the future.” In terms of the benefits from the training program, the participants in both groups reflected that knowledge of theory and practical learning activities by demonstration and self-practice under supervision, and feedback, were sufficient to enable them to manage the wheelchair for users in their community. In particular, they valued the fieldwork experience and teamwork, which gave them more confidence in transferring knowledge and learning from the training program to real situations. Key concepts in this theme were elicited from the transcripts and placed into categories that were explained in detail as follows.

1. Category 1: sufficiency of knowledge and practical learning to build confidence

The participants in both groups reflected that the “sufficiency of knowledge and practical leaning to build confidence” not only referred to learning activities in the training program but also included a manual of the training program that participants could access knowledge and understand. They provided feedback on how they received sufficient knowledge and practical learning, which they were able to use in real situations. Illustrative examples of statements from the elderly participants are as follows.

- Focus Group A:

FG-A-1: *‘The manual of the training program contained sufficiency of knowledge that could be followed during the theory and fieldwork experience sessions.'*

FG-A-3: *‘I feel proud of myself in that I can discuss areas of knowledge I learnt within my team and among others.'*

FG-A-4: *‘Before participating in the training program, I had no confidence in learning because of my old age. In contrast, after finishing the program, I think I now have enough knowledge to consider using the wheelchair, and the confidence to share my knowledge of wheelchairs with users in my village.'*

(ii) Focus Group B:

FG-B-3: *‘Before starting each new period of the theory session, we reviewed previous lessons with others, and it was not difficult to understand and recall the knowledge we had learnt.'*

FG-B-9: *‘Wheelchairs in the community had various types that were different from those we learnt about in the theory session. At first, we didn't think we could do it…. On the other hand, we worked together to consider each part….and can now apply our knowledge and practical experience in real situations.'*

(2) Category 2: values of the fieldwork experience

Participants in the fieldwork experience session performed their assignment with their groups and could provide services such as evaluating, providing suggestions, and adjusting to more suitability for individual users. The participants in both groups perceived the values of the fieldwork experience. However, each group focused on quite different values. The participants from Focus Group A reflected that the fieldwork experience had direct value for wheelchair users in the community. They were able to expand the community healthcare services for their wheelchair users in the fieldwork experience session. At the same time, the participants from Focus Group B focused on values of the fieldwork experience, which had impact on themselves. They expressed that the feeling of self-esteem rose after the fieldwork experience session. Illustrative examples of statements from the caregiver participants are as follows.

- Focus Group A

FG-A-2: ‘*The training program can encourage us to learn and develop, even though we may be elderly people or have a low education level…. It's also useful for wheelchair users in the community.'*

FG-A-4: *‘The fieldwork experience session is useful for elderly individuals, and people with disabilities in the community. An elderly individual who uses a wheelchair told me that although she frequently receives a home-visit service from primary healthcare providers in the community, they focus on health, nutrition and home environment…. Interestingly, she told me that this is the first time she received a follow up service after she was provided with a wheelchair. I felt proud that I was a part of her happiness.'*

(ii) Focus Group B

FG-B-10: *‘When I explained to a wheelchair user that the wheelchair armrests were inappropriate for his posture by causing him to shrug his shoulders, he refused to adjust because he thought he was familiar with that position. I motivated him to let me try and demonstrate and he allowed me…. He had shiny eyes when he said that he felt more comfortable. I cannot believe that I can use my knowledge to support others in a real situation.'*

FG-B-11: *‘It's unbelievable that I can evaluate, give suggestions of maintenance, and tighten the screw of the footrests. You know? I'm a woman and had no knowledge or experience of mechanics before…. An elderly individual who uses a wheelchair told me that he does not feel abandoned and is comfortable from taking this care as a community member. This makes me feel good.'*

FG-B-12: *‘There were two wheelchair users in my fieldwork experience. Both of them had never trained or received information on maintenance and adjustment of suitable wheelchairs for the user's body proportion. The fieldwork session brought service provision of community health via community members. Adjustment of wheelchairs can be done with general mechanical tools. After adjustment, wheelchairs are used with the appropriate position…. Before participating in this training program, I did not believe that I could learn and help others. It's an amazing feeling.'*

(3) Category 3: team support

The participants of both groups reflected the process of learning as a team in order to build confidence. In both the theory and fieldwork experience sessions, the participants were assigned to practice, plan, and manage the service with group work. Each group was set up by considering various backgrounds for sharing and supporting in order that an assignment succeeds. Illustrative examples of statements from the participants are as follows.

- Focus Group A

FG-A-3: *‘…. I worked with my group to evaluate the wheelchair, discuss the best suggestions and adjust some parts of the wheelchair for their users. The team made me feel comfortable and confident when we work in the community…. Besides sufficient knowledge and the working of wheelchair evaluation and modification in the community… ready willpower can be encouraged by the team group.'*

(ii) Focus Group B

FG-B-9: *‘We work together to consider each part of the wheelchair by evaluation and demonstration to modify the manual wheelchair to a power one. If I worked alone, I think I would be nervous. Group work is better….'*

In terms of improving a future training program, weaknesses should be considered such as perspectives of the participants that lack related-organizational support, which is unable to continue in the community or expand to other communities, as well as limited accessibility to the wheelchair database. Therefore, the participants suggested an informal option of contact between their working groups after they finish the training program. Key concepts in this theme also were placed into three categories such as “Organizational support”, “Expansion in various contexts,” and “System of continued connection and services after training.” Details of each category are as follows.

1. Category 1: organizational support

The participants of both groups reflected a problem in the training program, which was limited funding. Receiving support from related organizations, including government and private organizations, might be more effective. This support could be financial such as providing spare parts for wheelchairs and producing simplified power wheelchair sets and also giving manpower support, for example, connecting related professionals and specialists for sharing knowledge and providing services in complicated cases. Illustrative examples of statements from the participants are as follows.

- Focus Group A

FG-A-3: ‘*…I understand this is a limitation of this research, but if we have support from the municipality to produce more sets, we could increase the number of clients in the fieldwork experience session.*'

FG-A-1: *‘I agree with FG-A-3 that if we have more support from related government organizations, it would be better…. Private organizations are also interesting. We have the prototype that can improve our local market. The local government organizations might cooperate with local community entrepreneurs, such as machine garages, vehicle repair shops and so on.'*

(ii) Focus Group B

FG-B-1: ‘*Cost of producing simplified power wheelchair sets was not too expensive when compared to the price of a power wheelchair in a store. It's worth it for local government to support the elderly people in the community.'*

(2) Category 2: expanding various contexts

Perspectives of the participants indicated that adding more sessions in various contexts brought about better learning outcomes from the training program. This might reflect that although the participants were satisfied with the weight of knowledge content, they needed more fieldwork experiences in various situations. Moreover, each group focused on the differences in expanding various contexts. The participants from Focus Group A focused on expansion to other communities while the participants from Focus Group B focused on expanding to school settings in their community. Illustrative examples of statements from the participants are as follows.

- Focus Group A

FG-A-2: ‘… *it would be better to add a session of field trips in order to learn from other sources…and if possible, expand the fieldwork experience to other communities.'*

(ii) Focus Group B

FG-B-6: ‘…*in this district we have a special education school for students with physical disability. We should expand the training program to cover wheelchair services for these children. There are a lot of wheelchairs for practice and the children would gain advantage by receiving the services.'*

(3) Category 3: system of continued connection and services after training

The system of linking the training program of the community learning center to related service providers could be synthesized by the perspectives of the participants and be a way of collaborating between elderly people and caregivers, who were trained from the community learning center, health service providers at the local community hospital, and the municipality. In addition, because the elderly and caregivers were not formal officers of the hospital or municipality, they could not access the database of stored wheelchairs and wheelchair users in the community. Moreover, the participants were from different villages; therefore, it might be difficult to share information and request help. The participants from Focus Group A suggested formal and informal options of the connection system. In particular, the participants from Focus Group B suggested specifically using a social network application (LINE application) as a connecting tool. Illustrative examples of statements from the participants are as follows.

- Focus Group A

FG-A-2: *‘The training program should be continued because it is useful for everyone who lives in the community…. a support system from community organizations, both formal and informal, will bring about sustainability of this service after training.'*

(ii) Focus Group B

FG-B-9: ‘*This training program has great impact on our community, but it doesn't have the system to connect us with a typical service in the community…if it has a channel or system, for example… using a social network application...a LINE group… that we can join after this program has finished, it would be awesome…*.'

Conclusion of the themes and categories in evaluating the training program by focus group discussion, as described above, is shown in Table 2.

### 3.3. Result of the Wheelchair Evaluation and Service Provision for Wheelchair Users

Seventeen wheelchair users in the community agreed to be evaluated during the fieldwork experience session. Most of the users (70.59%) had more than one mobility aid and used wheelchairs both in and around the home (58.82%), as well as only in the home (41.18%), when having no use for them outside. This might be because the environment in their community had barriers for the use of wheelchairs outside the home such as thoroughfares with uneven surfaces and no bicycle or wheelchair paths, which could be dangerous. In addition, although most wheelchairs were in good condition (82.35%), it was found that footrests had the highest percentage for inappropriateness (41.18%). Besides the participants being able to gain their experience for wheelchair evaluation during this session, the wheelchair users received service from the participants. The results indicated that after evaluation the participants provided suggestions of maintenance for wheelchair users (76.47%) and adjustments to inappropriate parts for individual users (29.41%).

## 4. Discussion

In this study, the prototype was an important tool in the training program. This was because most of the learning participants in this study were elderly, and none of them had experience of wheelchairs or other assistive devices or technologies. The prototype was modified from equipment in Thai markets, with consideration given for the characteristics and limitations of the learners. In addition, all parts of the prototype were separated and could be connected by an electric wire set. The reason for designing three separate parts was that if one were to break, the user could change that part by themselves and save their time and money. Furthermore, the local government or subdistrict municipality could take each part of the prototype to local stores or local entrepreneurs for producing them for the community. It is important that regarding the background and limitation of the elderly and caregiver learners, the training program focused on their ability to learn how to assemble a manual wheelchair for evaluation and modification without them needing advanced mechanical skills.

In the training program phase, adult learning is a crucial component of the continuous learning concept in order that the characteristics of elderly learners are considered a step in designing the training program. Moreover, a successful learning program should be considered for gaining confidence in a program that could enhance knowledge, skills, and abilities for elderly learners [24]. Development of the training program for the elderly and caregiver learners was new in the Thai context. This was based on the view of learning that can occur through life or long-life. There were two main points in the stage of developing the program such as teaching methodologies and areas of learning activities. First, the teaching methodologies were different from teaching young students because adult learners have different needs and motivations for learning. In other words, their learning activities were different from those for young learners. Thus, researchers, as lecturers in the training program, should take on the role of facilitators, not teachers, in order to motivate participation, discussion, and challenges in new lessons, because a successful training program for elderly learners needs their involvement [25–27]. Second, the areas of learning activities should include basic learning, which involves general knowledge, integration of necessary knowledge and skills to complete tasks, and social learning [27]. Complicated theory and multistage learning activities might not encourage the elderly or caregivers to succeed or meet learning outcomes. Besides knowledge, practical experience, and social skills, these activities were expected to promote the elderly and caregivers to contribute actively to their communities as potential community members. Therefore, this training program was developed to cover general knowledge and encourage experience in real situations by group working, with the expectation of relating to learning styles and activities for elderly people. In the hours of practice, the participants learned various activities. Presentation was the most challenging activity for the participants. Most of them were not familiar with speaking into a microphone when standing in front of the class. However, they were encouraged to meet this challenge and consequently improved with group support and freedom to design their own presentation. These relaxed and flexible approaches enabled support for learning opportunities, with a sense of achievement that was an important factor in an effective training program for elderly learners [28]. In summary, these learning and practical achievements in the training program were able to increase worthiness for the elderly and their caregivers as service providers at the primary care level in their community. As adult learners, particularly the elderly, they not only gained skills by working in group activities but also developed or recovered their leadership and collaborative behavior to reach successful outcomes [29].

Linking the training program of the community learning center to related service providers was an important point. It reflected the perspectives of the participants on continuity of the training program and pushed it towards being part of the community learning center. In fact, community healthcare services in suburban areas were at the primary care level [8]. Elderly individuals were provided with services for basic care by a local community hospital that lacked specialists, including wheelchair specialists [12, 13]. Many community learning centers in Thailand were set up recently as schools for village health volunteers and others for the elderly. The purpose of setting up schools for village health volunteers was to encourage community volunteers to take care of their community members by working with health professionals from local community hospitals. The purpose of setting up schools for the elderly in communities was to support social participation and maintain functions, including those that are physical and mental. Thus, having a training program in the community learning center could be a way of enhancing the potential of village health volunteers, elderly people, and caregivers. However, as the participants were not formal officers of the hospital or municipality, using a social network application was considered to be a connection tool. It was a guideline that not only merely proposed relating to an organization in the community but also was meant as an action for stakeholders to offer the needs and hear the perspectives of the participants. This related to the WHO [30] in promoting the setup of a concrete system for health services in communities by considering the entry of cultural dimensions.

In making the point of improvement, the elderly people in this study paid importance to government support in continuing the training program and expanding the training duration. This reflected the lack of government or business involvement as one of the barriers against sustainability in elderly learners [31]. In addition, the requirement of a longer training program related to the learning styles of elderly learners, which motivated learning from experience [28] that enhanced their confidence and self-esteem by actively participating. Furthermore, most elderly individuals in suburban communities in Thailand have less opportunity to use high-tech devices in performing their daily activities [4]. Therefore, they might need more time to familiarize themselves with these technologies.

A limitation of this study was the generalization of different contexts. This study operated in suburban villages that had specific conditions. In addition, it could only develop one prototype for sharing among the groups of participants in the training program, due to the limitation of research funding. Therefore, the number of wheelchair users and areas was limited in the step of the fieldwork experience session, in which the participants were assigned to evaluate and demonstrate the modification of only manual wheelchairs that had owners. On the other hand, there were many broken wheelchairs in the storeroom of the local community hospital and disused wheelchairs in the village health center that were not assigned to the fieldwork experience session. Thus, the experience received by the participants was limited in using-status, and most of the wheelchairs were in usable condition. These factors are of interest in expanding the opportunity of more experience in the fieldwork experience session for elderly people and caregivers. In addition, although this study focused on encouraging healthy elderly people and their caregivers to be potential community members and contribute their knowledge and experience to taking care of wheelchair users in their community, feedback from the end users was not measured systematically. Exploring improvement of quality of life for wheelchair users in the community, via the contribution of healthy elderly people and their caregivers, is an interesting point for future research. This would support basic health services for dependent community members, who depend on or wait for only specialists, who are insufficient in number for providing a service to communities.

## 5. Conclusion

This study is aimed at developing a training program to evaluate and modify the manual wheelchair to a simplified power wheelchair for elderly people and their caregivers in the community, in the belief of potential life-long learning for elderly individuals. This was a new approach to healthcare promotion services in the communities of Thailand to promote community health by community members. However, it also could be a guideline for developing new training programs by considering sufficiency of knowledge and practical learning and opportunities of gaining fieldwork experience in various contexts, as well as encouraging teamwork and systematically continuing governmental and private organizational support. In addition, this study could reflect on local policy makers as the starting point of a wheelchair service center for providing services and sharing resources among community networks, in which the elderly and their caregivers play a mentoring role in this pilot area for support to more communities.

## Figures and Tables

**Table 1:** Characteristics of the participants (*n* = 24).

Characteristics	Numbers (percentage)
Gender	
Male	18 (75.00)
Female	6 (25.00)
Age (years)	
30–39	1 (4.17)
40–49	3 (12.50)
50–59	5 (20.83)
60–69	13 (54.17)
70–79	2 (8.33)
Education level	
Uneducated	2 (8.33)
Primary education level	7 (29.17)
Secondary education level	8 (33.33)
High school level	6 (25.00)
Bachelor's degree	1 (4.17)
Average income (baht/month)	
Less than 3,000	12 (50.00)
3,000–5,000	4 (16.67)
More than 5,000	8 (33.33)
Representative status in the training program	
Elderly people	12 (50.00)
Elderly people who are also family caregivers	3 (12.50)
Community volunteer caregivers	9 (37.50)
Experience regarding wheelchair or any other mobility aids	
Yes	0 (0.00)
No	24 (100.00)

**Table 2:** Evaluation of the training program by focus group discussion.

Themes	Categories
Benefits from the training program	(1) Sufficiency of knowledge and practical leaning to build confidence
	(2) Values of fieldwork experience
	(3) Team support
Improvement of the training program in the future	(1) Organizational support
	(2) Expansion in various contexts
	(3) System of continued connection and services after training

## Data Availability

The data used to support the findings of this study are included within the article and any further data can be provided from the corresponding author upon request.
